# Effect of TiN Interfacial Layer on the Microstructure and Optoelectronic Properties of AZO/Ag/AZO Multilayer Films

**DOI:** 10.3390/nano16171103

**Published:** 2026-09-01

**Authors:** Haijuan Mei, Yi Yu, Libin Gan, Rui Wang, Zhaohui Guo, Qiuguo Li, Hongping Liang, Huojuan Ye, Zhenting Zhao, Weiping Gong

**Affiliations:** 1 Guangdong Provincial Key Laboratory of Electronic Functional Materials and Devices, Huizhou University, Huizhou 516007, China; 2 Guangxi Key Laboratory of Special Engineering Equipment and Control, Guilin University of Aerospace Technology, Guilin 541004, China

**Keywords:** AZO/Ag/AZO, TiN, microstructure, optoelectronic properties

## Abstract

To investigate the effect of the TiN interfacial layer on the microstructure and optoelectronic properties of AZO/Ag/AZO multilayer films, four configurations, namely AAA, ATAA, AATA, and ATATA, were deposited on the glass substrates by magnetron sputtering. All the films exhibited a preferential ZnO (002) orientation. When TiN was located above the Ag layer, the ATAA film preserved a pronounced Ag (111) diffraction feature and exhibited the best overall optoelectronic performance, the average transmittance increased from 78.4% for AAA to 85.9%, the resistivity decreased from 7.2 × 10^−5^ to 6.3 × 10^−5^ Ω·cm, and the figure of merit increased from 0.64 to 0.71 Ω^−1^. In contrast, when Ag was grown directly on TiN, the Ag (111) diffraction peaks of the AATA and ATATA films were markedly weakened, the average transmittance decreased to 59.0% and 62.0%, respectively, and the resistivity increased sharply to 1.2 × 10^−3^ and 9.5 × 10^−4^ Ω·cm, respectively. These results demonstrate pronounced interfacial asymmetry and stacking-sequence dependence in the TiN regulation of AZO/Ag/AZO films, with the ATAA configuration exhibiting the best structure–optics–electronics synergy.

## 1. Introduction

Transparent conductive films combine high visible-light transmittance with efficient carrier transport and are key functional materials in optoelectronic devices such as solar cells, flat-panel displays, light-emitting diodes, and flexible electronics. Conventional indium tin oxide (ITO) exhibits excellent transparent-conducting performance. However, the limited availability and high cost of indium, together with the mechanical brittleness of ITO films, restrict its further use in low-cost and flexible devices. In comparison, Al-doped ZnO (AZO) is considered an important indium-free transparent-conducting candidate because of its abundant raw materials, low cost, environmental compatibility, and high visible-light transmittance [1]. Nevertheless, the resistivity of single-layer AZO films is still insufficient for some high-performance transparent-electrode applications. To alleviate the trade-off between optical transparency and electrical conductivity, the use of a transparent conductive oxide/metal/transparent conductive oxide (TCO/metal/TCO, TMT) multilayer structure, in which an ultrathin metal layer is sandwiched between two TCO layers, has become an important strategy for improving overall optoelectronic performance [2,3]. Owing to its extremely low intrinsic resistivity and favorable optical properties in the visible range, Ag has been widely used in AZO/Ag/AZO transparent electrodes [4,5,6,7]. However, when the Ag layer is reduced to the nanometer scale, its continuity becomes highly sensitive to the deposition interface. Moreover, Ag may undergo diffusion and agglomeration during thermal treatment or long-term service, leading to degradation of the interfacial structure and optoelectronic properties.

At the nanoscale, Ag film deposition generally proceeds through nucleation, island growth, and island coalescence. The nucleation density, wettability, and cluster-coalescence behavior directly determine the critical thickness at which Ag evolves from discrete islands into a continuous or quasi-continuous conductive layer, thereby affecting carrier scattering, optical absorption, and interfacial reflection. Consequently, interface engineering has become an important route for optimizing Ag-based TMT transparent electrodes. Previous studies have shown that controlling the oxide surface state and interfacial energy, or introducing seed layers and mild oxidation treatments, can effectively improve the nucleation and continuous-film formation of ultrathin Ag [8,9,10,11]. On this basis, ultrathin Ti, Ni, and Al interfacial layers have been employed to regulate metal/TCO interfaces. Zhu et al. prepared AZO/Ti/Ag/AZO films and reported that the insertion of a 1 nm Ti layer reduced the surface roughness, increased the carrier mobility, and improved near-infrared transmission [12]. Ji et al. further demonstrated that a sub-nanometer Ti wetting layer enhanced Ag adhesion on oxide surfaces and stabilized early-stage Ag nanoclusters, thereby promoting the formation of thinner, more continuous, and smoother Ag layers [13]. Regarding interfacial stability, a Ni interlayer can effectively suppress Ag diffusion and oxidation, allowing AZO/Ni/Ag/AZO films to retain low sheet resistance and structural stability after thermal oxidation at 500 °C [14]. Al interfacial layers can likewise restrict Ag diffusion into AZO and markedly improve the thermal stability of AZO/Al/Ag/Al/AZO electrodes [15]. Similar interface-engineering concepts have also been extended to Cu-based TMT systems, including AZO/Ti/Cu/AZO [16,17], AZO/Al/Cu/AZO [18], WZO/Al/Cu/Al/WZO [19], and WZO/Cr/Cu/Cr/WZO [20] films.

Compared with these metallic interfacial layers, titanium nitride (TiN) combines electrical conductivity, good thermochemical stability, and a pronounced free-carrier optical response in the visible to near-infrared range. Its electrical and optical properties are also strongly dependent on film thickness and microstructure [21,22,23], making TiN a potential functional interfacial layer for Ag-based transparent electrodes. Gao et al. deposited Ag/TiN films on Si substrates and showed that a 12 nm TiN diffusion barrier enabled the Ag/TiN/Si structure to maintain stable sheet resistance after annealing at 600 °C [24]. However, the Ag and TiN thicknesses and the substrate system used in that study differ substantially from those of nanoscale AZO/Ag/AZO transparent electrodes. The distinct effects of positioning ultrathin TiN at the interface formed before Ag deposition, at the interface formed after Ag deposition, or on both sides of Ag have not yet been systematically compared. In this study, the AZO/Ag/AZO, AZO/TiN/Ag/AZO, AZO/Ag/TiN/AZO, and AZO/TiN/Ag/TiN/AZO films were deposited. The effects of TiN position and interfacial configuration on the microstructure and optoelectronic properties were systematically examined to clarify the stacking-sequence-dependent regulation by TiN and to provide an experimental basis for interface-structure optimization in Ag-based TMT transparent conductive films.

## 2. Experimental Details

### 2.1. Film Deposition

**Figure 1:**
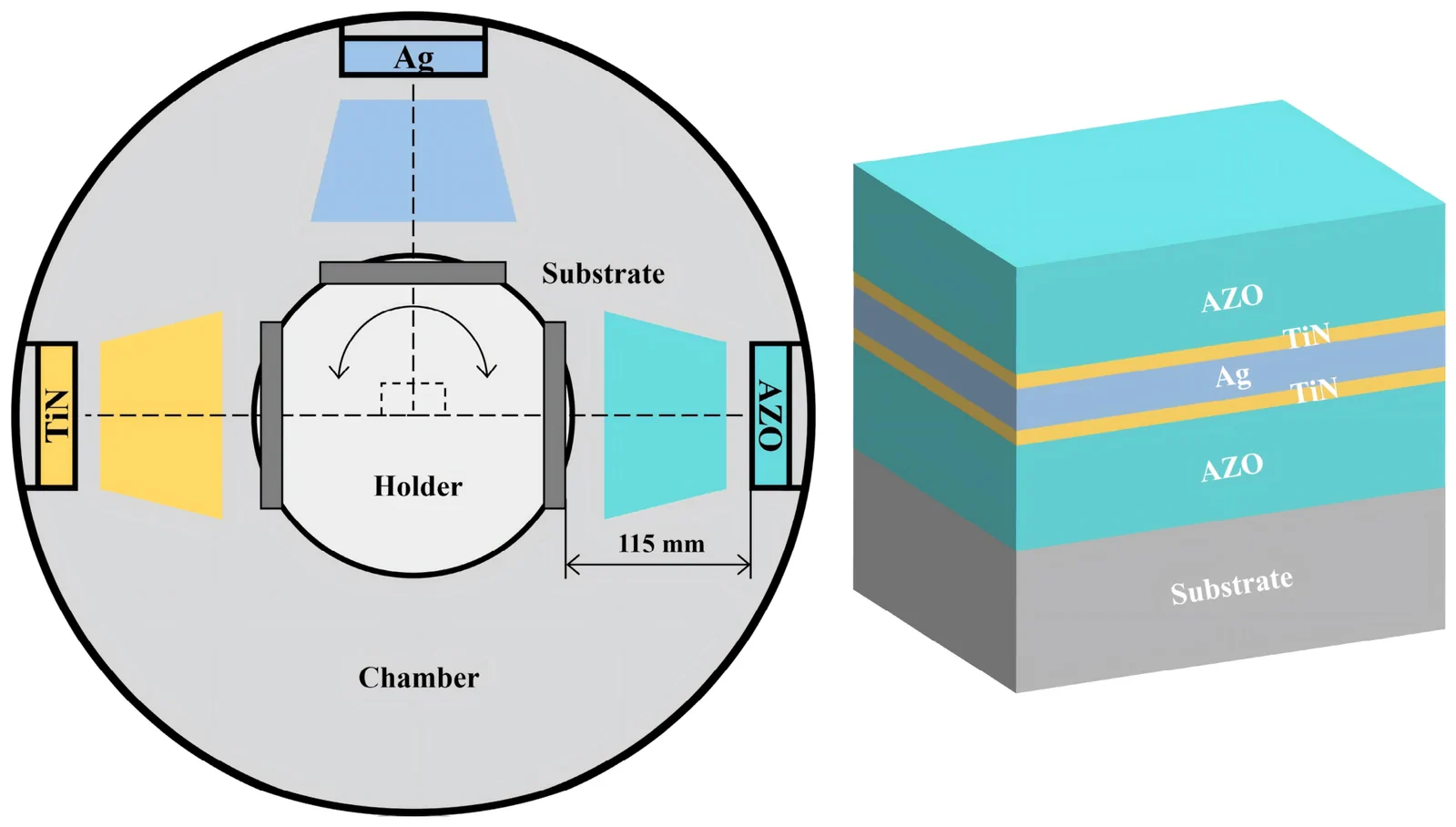
Schematic diagram of multilayer film deposition.

The AZO/Ag/AZO (AAA), AZO/TiN/Ag/AZO (ATAA), AZO/Ag/TiN/AZO (AATA), and AZO/TiN/Ag/TiN/AZO (ATATA) multilayer films were deposited on conventional glass substrates by radio-frequency (RF) and direct-current (DC) magnetron sputtering. The targets were AZO (ZnO:Al_2_O_3_ = 98:2 wt%, 99.9% purity), Ag (99.99% purity), and TiN (99.9% purity), all with dimensions of Ø76.2 mm × 4.0 mm. The multilayer films were deposited using the sputtering system illustrated in Figure 1. Before deposition, the glass substrates were ultrasonically cleaned successively in acetone and absolute ethanol for 20 min, dried, and fixed on the substrate holder. The chamber was evacuated to a base pressure of 8.0 × 10^−4^ Pa using a mechanical pump and a molecular pump, after which high-purity Ar was introduced to regulate the working pressure. Both the bottom and top AZO layers were deposited by RF magnetron sputtering at 300 W and 1.5 Pa for 5 min. The intermediate Ag layer was deposited by RF magnetron sputtering at 90 W and 1.5 Pa for 124 s. The TiN interfacial layer was deposited by DC magnetron sputtering at 100 W and 0.5 Pa for 28 s. Before TiN deposition, the TiN target was pre-sputtered with the substrate shutter closed until the discharge and sputtering process reached a stable state. The detailed deposition parameters are listed in Table 1. The deposition sequences for ATAA and AATA were bottom AZO → Ag → TiN → top AZO and bottom AZO → TiN → Ag → top AZO, respectively. Thus, TiN in ATAA film is located at the upper interface formed after Ag deposition, whereas TiN in AATA film is located at the lower interface formed before Ag deposition. The nominal layer thicknesses calculated from the thick-film deposition-rate calibrations and deposition times are summarized in Table 2.

### 2.2. Film Characterization

The surface and cross-sectional morphologies of the films were examined using a scanning electron microscope (SEM, Vega3, Tescan, Brno, Czech Republic), and the thicknesses were determined from the cross-sectional images. The phase structures of the films were characterized by X-ray diffraction (XRD, MiniFlex 600, Rigaku, Akishima, Japan) in a conventional θ–2θ configuration. After Gaussian fitting of the diffraction peaks, the grain size, lattice constant, and residual stress were calculated using the Scherrer equation [25], Bragg’s law [26], and a biaxial strain model [27], respectively. Optical transmittance over 350–1000 nm was measured using a transmittance spectrophotometer (723PCSR, Ruifeng, Guangzhou, China). The resistivity, carrier concentration, and Hall mobility of the films were measured at room temperature using a Hall-effect measurement system based on the van der Pauw method (CH-100, Cuihai, Beijing, China). For the optical transmittance and Hall parameters, each sample was measured at least three times.

## 3. Results

### 3.1. Microstructure

**Figure 2:**
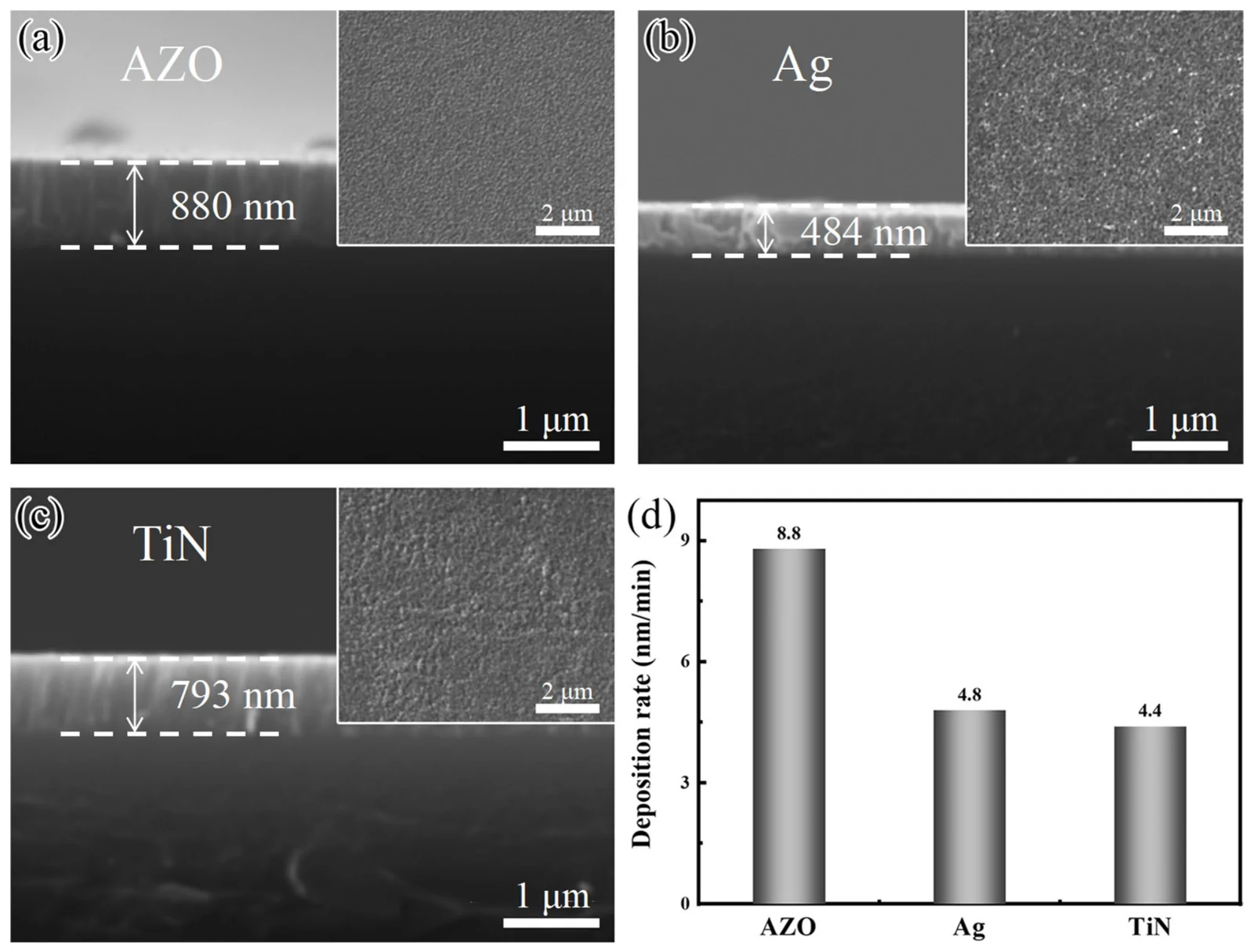
Surface and cross-section SEM images, deposition rate of the monolayer films: (**a**) AZO, (**b**) Ag, (**c**) TiN, (**d**) deposition rate.

Figure 2 presents the surface and cross-sectional SEM images and the corresponding deposition rates of the AZO, Ag, and TiN monolayer films. The AZO film consists of relatively uniform fine grains and exhibits a smooth, dense surface, while its cross section shows a pronounced columnar-growth morphology, and the calibrated thickness is approximately 880 nm. The Ag film exhibits a more undulating and granular surface, with a calibrated thickness of approximately 484 nm. Ag film deposition generally proceeds through initial nucleation, island growth, and island coalescence, and an ultrathin Ag layer gradually develops into a continuous or quasi-continuous conductive film only after a critical thickness is reached [9,10,11]. In contrast, the TiN film is composed of finer surface grains and forms a relatively continuous and dense layer, with a cross-sectional thickness of approximately 793 nm. It should be noted that the morphologies of the three monolayer films are jointly affected by the intrinsic material properties, sputtering power, working pressure, and deposition thickness. Based on the measured cross-sectional thicknesses and deposition times, the deposition rates of AZO, Ag, and TiN were calculated to be 8.8, 4.8, and 4.4 nm/min, respectively. In our previous studies on related AZO/Al/Cu/AZO and WZO/Cr/Cu/Cr/WZO multilayer systems [18,20], the layer and total thicknesses estimated using the same rate-time method were in good agreement with cross-sectional TEM measurements. This agreement supports the use of the rate-time method as a practical approach for estimating nominal layer thicknesses under related deposition conditions. Because the AZO, Ag, and TiN layers in the actual multilayer structures are all nanoscale, the thick monolayer films in Figure 2 were used primarily for deposition-rate calibration. Accordingly, the 44 nm AZO, 10 nm Ag, and 2 nm TiN layers in the films are nominal thicknesses calculated from the deposition rates, as summarized in Table 2.

**Figure 3:**
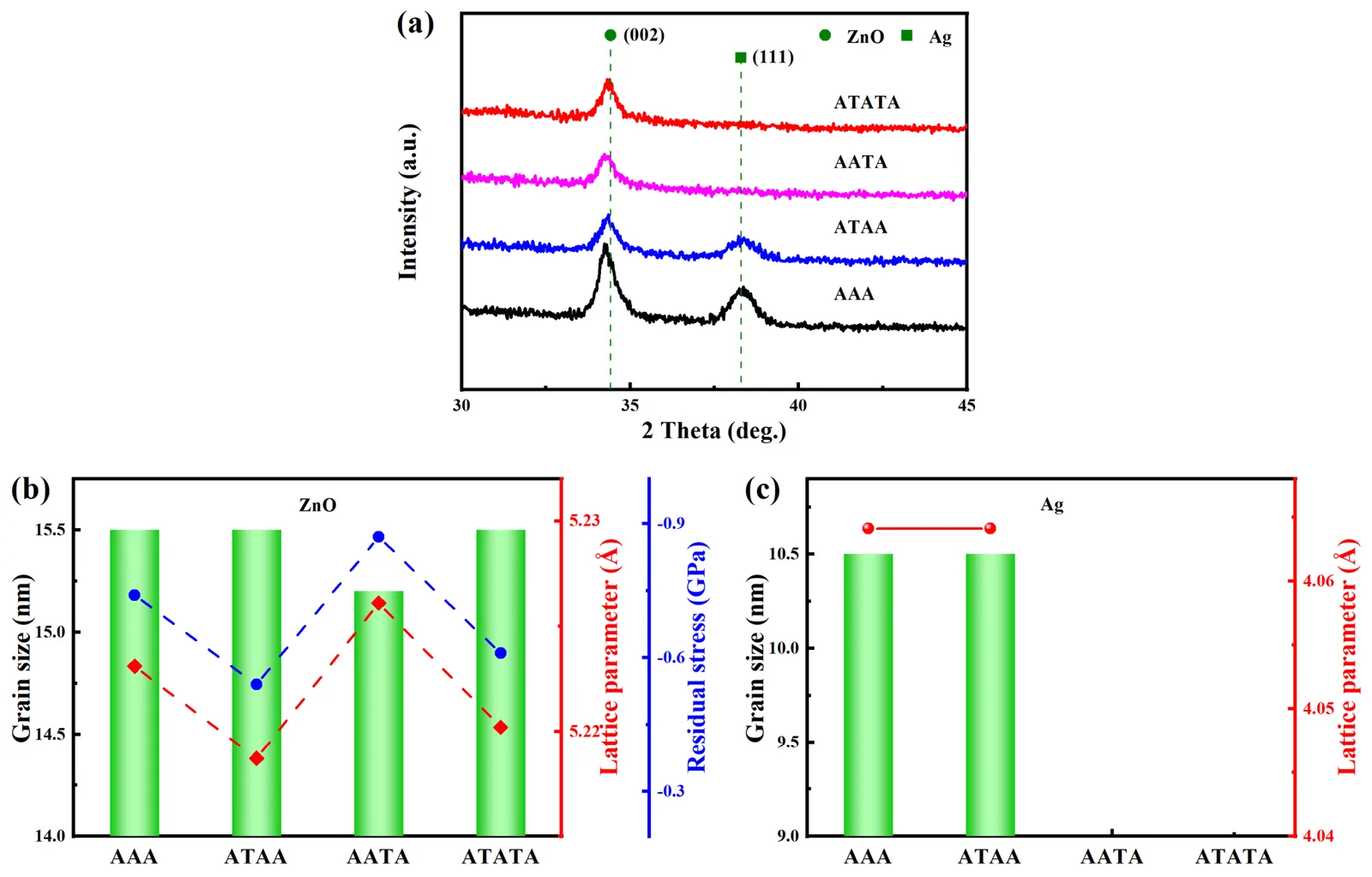
(**a**) XRD pattern of the multilayer films, (**b**) grain size, lattice parameter, and residual stress of the AZO films, (**c**) grain size and lattice parameter of the Ag films.

Figure 3a shows the XRD patterns of the multilayer films. All the films exhibit a pronounced ZnO (002) diffraction peak near 34.3°, indicating that the AZO layers retain the hexagonal wurtzite structure and a strong c-axis preferred orientation under different TiN interfacial configurations. In contrast, the Ag (111) diffraction response is much more sensitive to the TiN position. Distinct Ag (111) peaks are observed near 38.3° for AAA and ATAA, whereas the corresponding peaks in AATA and ATATA are markedly weakened and become difficult to distinguish. Considering the actual deposition sequence, Ag in both AAA and ATAA was deposited directly on the bottom AZO layer, so the two structures share the same initial Ag nucleation substrate. Moreover, the Ag (111) grain sizes in both films are approximately 10.5 nm, indicating that the 2 nm TiN layer deposited after Ag does not significantly alter the grain size or preferred orientation of the preformed Ag layer. In AATA and ATATA, TiN was first deposited on the bottom AZO layer, followed by Ag nucleation and growth on TiN. Ag (111) peak intensity may be affected by texture, preferred orientation, coherent diffraction volume, and crystalline ordering. Consequently, the weakened Ag (111) peaks observed for AATA and ATATA indicate altered Ag structural ordering. No resolvable TiN diffraction peak is observed for any sample, mainly because the TiN layer is only 2 nm thick and may exist in an amorphous or very fine nanocrystalline state [18]. As shown in Figure 3b, the grain sizes calculated from the ZnO (002) peak are 15.2–15.5 nm for all four films, indicating that TiN position has only a minor effect on the AZO grain size. Nevertheless, the ZnO (002) peak position, lattice constant, and residual stress vary with the stacking sequence. ATAA exhibits relatively low compressive residual stress, which may be associated with interfacial coverage after Ag deposition, subsequent growth of the top AZO layer, and altered interlayer stress transfer rather than with modulation of the initial Ag nucleation. By contrast, the higher compressive residual stress in AATA may be related to the new interfacial constraint and structural mismatch created when Ag grows directly on TiN.

### 3.2. Optoelectronic Properties

**Figure 4:**
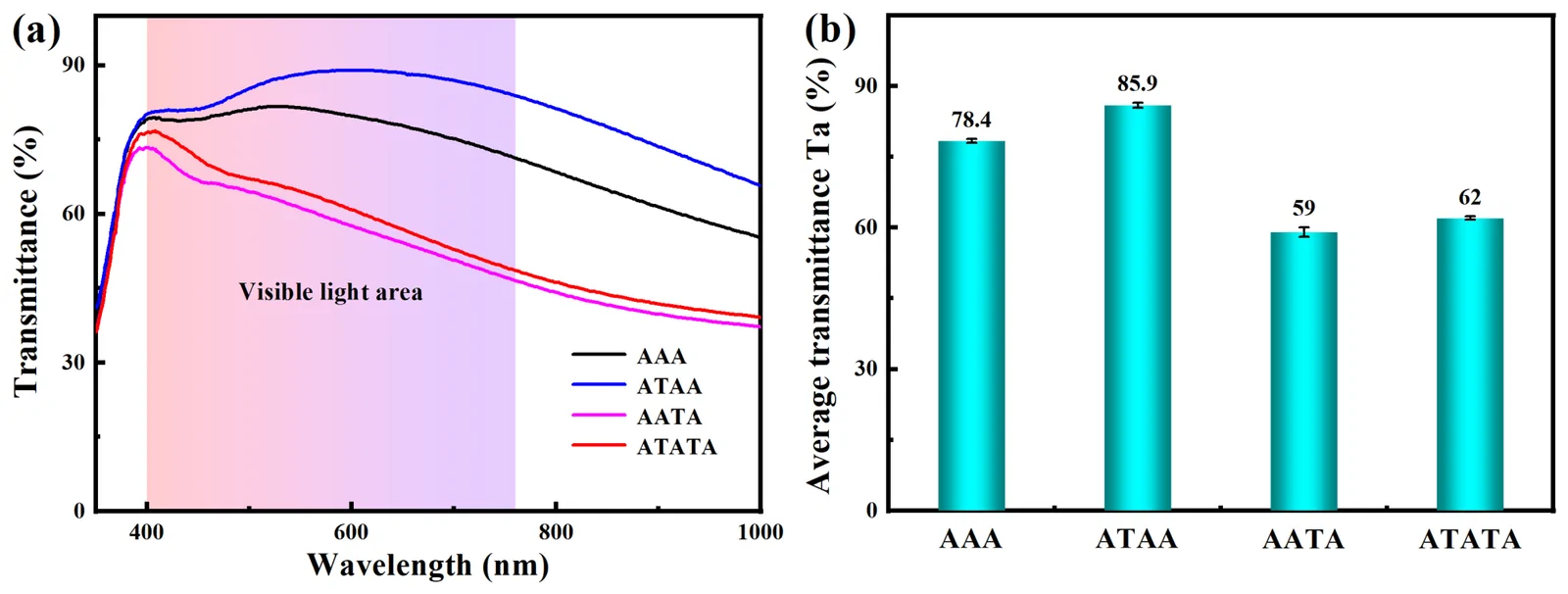
(**a**) Transmittance spectra, (**b**) average transmittance of the multilayer films.

Figure 4a presents the transmittance spectra of the AAA, ATAA, AATA, and ATATA films over 350–1000 nm, and Figure 4b shows their average visible-light transmittance in the 400–760 nm range. The average transmittance of AAA film is 78.4%. When TiN is deposited above the preformed Ag layer, the average transmittance of ATAA increases to 85.9% and remains higher than that of AAA over most of the visible spectrum. The optical response of a TMT multilayer is not a simple superposition of the transmittances of the individual layers, rather, it is jointly governed by metal absorption, interfacial reflection, and multilayer optical interference [6,7]. Therefore, the enhanced transmittance of ATAA cannot be attributed simply to the transparency of TiN itself. Because Ag in ATAA was deposited directly on the bottom AZO layer, its initial nucleation conditions are essentially the same as those in AAA. In addition, the Ag (111) grain sizes of the two films are nearly identical, indicating that the optical improvement does not originate from a change in the initial Ag nucleation or a significant increase in the Ag grain size. The average visible-light transmittance increases from 78.4% for AAA to 85.9% for ATAA, in which TiN was deposited above the preformed Ag layer. This measured increase is associated with the modification of the upper Ag interface after introducing the nominally 2 nm TiN layer. Changes in interfacial reflection, absorption, and multilayer interference may contribute to the observed transmittance enhancement. In contrast, the average transmittances of AATA and ATATA decrease to 59.0% and 62.0%, respectively. Notably, ATAA and AATA films have the same TiN thickness and total nominal thickness, yet their average transmittances differ by 26.9%, further demonstrating the pronounced effect of the asymmetric TiN/Ag and Ag/TiN interfaces. In both AATA and ATATA, Ag nucleates directly on TiN layer, and the Ag (111) diffraction peak is markedly weakened, indicating changes in Ag structural ordering and growth state. Hence, the reduced transmittance of these films is more reasonably attributed to the combined effects of altered Ag growth on TiN and additional interfacial optical losses. When an additional TiN layer is introduced above Ag in ATATA, the average transmittance recovers slightly relative to AATA but remains much lower than those of AAA and ATAA, suggesting that TiN above Ag layer provides partial optical compensation that is insufficient to fully offset the adverse effect of Ag growth on TiN.

**Figure 5:**
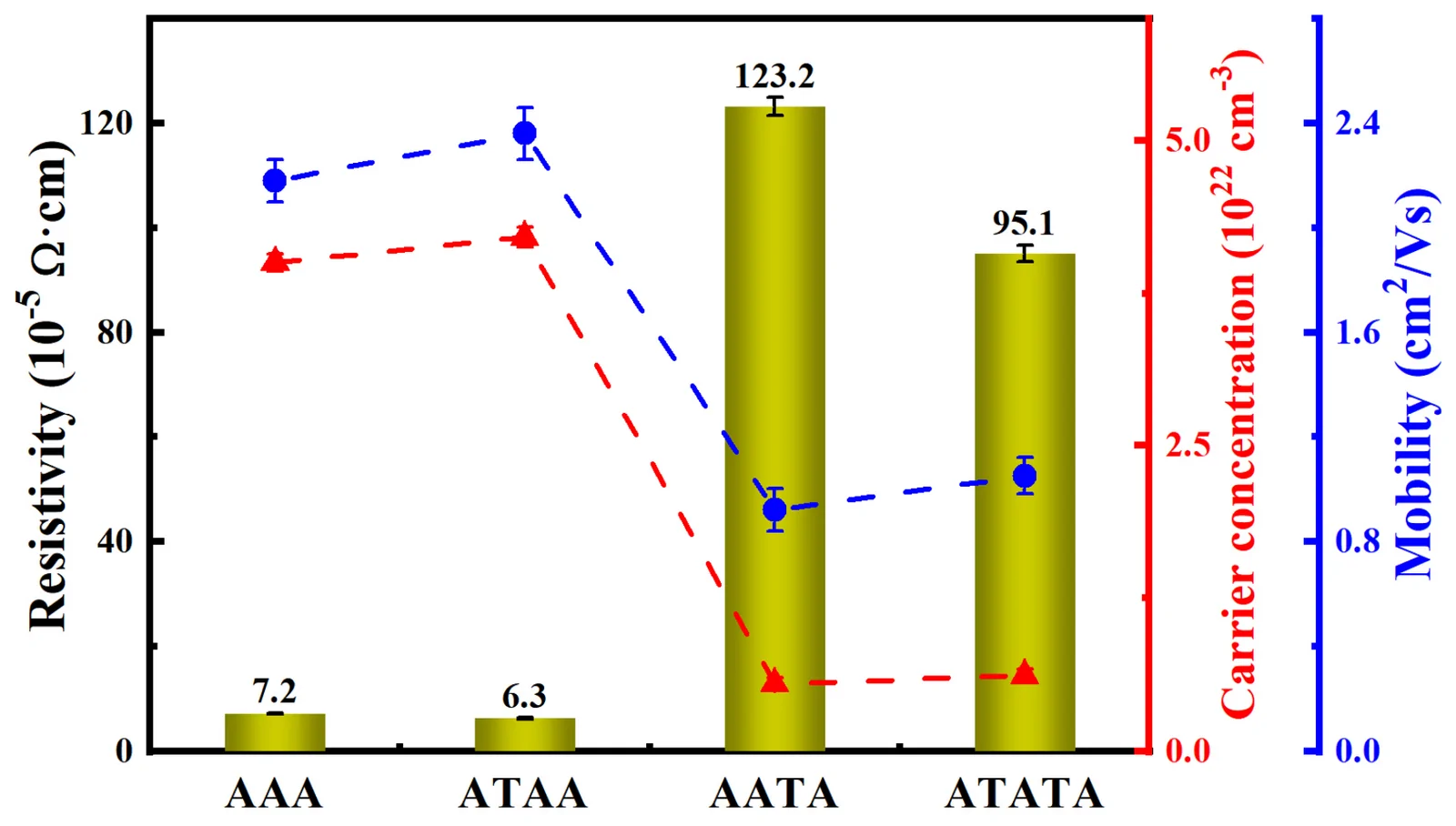
Measured resistivity, effective carrier concentration, and effective mobility of the films.

Figure 5 shows the measured resistivity, effective carrier concentration, and effective Hall mobility of the films. The resistivity of AAA film is 7.2 × 10^−5^ Ω·cm, while the carrier concentration and Hall mobility are 4.0 × 10^22^ cm^−3^ and 2.18 cm^2^·V^−1^·s^−1^, respectively. When TiN is introduced above the Ag layer, the resistivity of ATAA decreases to 6.3 × 10^−5^ Ω·cm, accompanied by simultaneous increases in carrier concentration and Hall mobility. For practical electrode comparison, the electrical properties are discussed primarily in terms of sheet resistance. The sheet resistance is calculated as *R_s_* = *ρ*/*t*, where *ρ* and *t* represent the resistivity and the nominal total thickness of the multilayer film, respectively. The sheet resistances of AAA, ATAA, AATA, and ATATA are approximately 7.3, 6.3, 123.2, and 93.2 Ω/sq, respectively. The ultrathin TiN layer modifies the upper surface state of Ag and creates a new AZO/TiN/Ag interface, thereby regulating interfacial defects and carrier-scattering processes and reducing the transport resistance across the multilayer structure [12,13]. In addition, charge transport in ultrathin TiN is highly sensitive to film thickness and defect state [22,23], and its contribution to the overall conduction path may also contribute to the improved effective carrier transport of ATAA. Accordingly, the reduced sheet resistance of ATAA can be attributed to the synergistic optimization of carrier scattering at the upper Ag interface and the effective transport channels of the multilayer film. In contrast, the resistivity of AATA increases markedly to 1.2 × 10^−3^ Ω·cm, while its carrier concentration and Hall mobility decrease to 5.5 × 10^21^ cm^−3^ and 0.92 cm^2^·V^−1^·s^−1^, respectively. When TiN is introduced on both sides of Ag layer, the resistivity of ATATA decreases to 9.5 × 10^−4^ Ω·cm, and both carrier concentration and Hall mobility show a partial recovery. The higher sheet resistances of AATA and ATATA coincide with the weakened Ag (111) diffraction features and indicate poorer in-plane electrical transport when Ag is deposited on the TiN-containing lower interface. These observations are consistent with altered Ag structural ordering and increased interface-related carrier scattering. Compared with AATA, ATATA contains an additional TiN layer above Ag and hence an additional upper Ag/TiN interface. Its lower resistivity and slightly higher carrier concentration and Hall mobility suggest that this upper-interface TiN provides a partial compensating effect on carrier transport, although it cannot fully offset the adverse effect associated with Ag growth on TiN. This further demonstrates that the effect of TiN on the electrical properties of the multilayers exhibits pronounced interfacial asymmetry and stacking sequence dependence.

**Figure 6:**
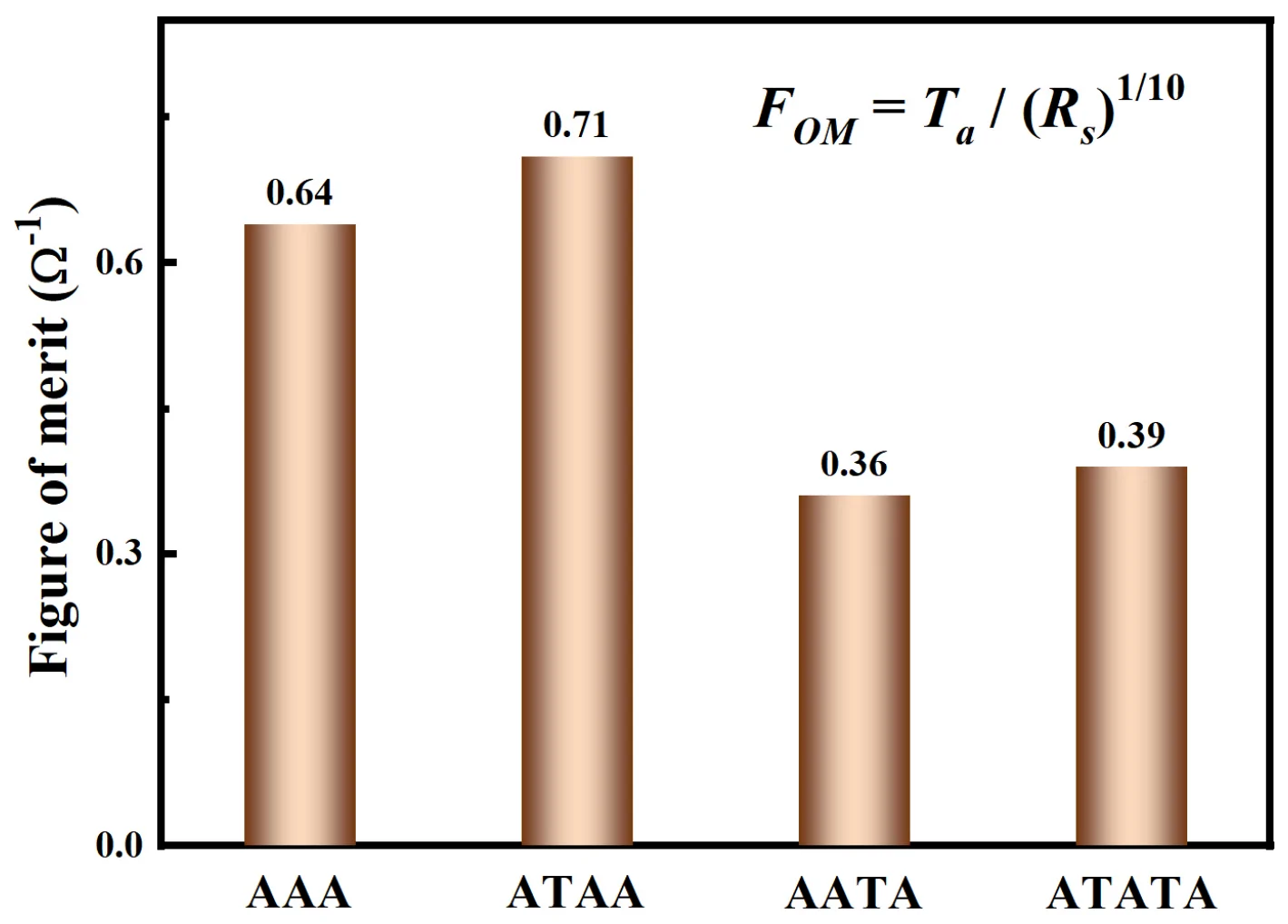
Figure of merit of the multilayer films.

To comprehensively evaluate the optical and electrical performance of the films, the high-resolution Haacke figure of merit (*F_OM_*) was calculated as *F_OM_* = *T_a_*/*R_s_*^(1/10)^ [28,29], where *T_a_* is the average transmittance over 400–760 nm and *R_s_* is the sheet resistance. The calculated *R_s_* values are 7.3, 6.3, 123.2, and 93.2 Ω/sq for AAA, ATAA, AATA, and ATATA, respectively. As shown in Figure 6, the *F_OM_* of AAA film reaches 0.64 Ω^−1^, indicating good optoelectronic properties and a performance level comparable to those reported for the AZO/Cu/AZO [18] and AZO/Ag/AZO films [30,31]. When TiN is deposited above Ag, the *F_OM_* of ATAA increases to a maximum value of 0.71 Ω^−1^, demonstrating simultaneous enhancement of transmittance and reduction in sheet resistance. Because Ag in ATAA still nucleates and grows directly on the bottom AZO layer, the Ag (111) preferred orientation and grain size remain almost unchanged relative to AAA, the performance enhancement does not originate from changes in the initial Ag nucleation or grain size. It is mainly associated with the regulation of the upper Ag optical boundary and carrier-scattering conditions by the ultrathin TiN capping layer deposited after Ag deposition. The resulting AZO/TiN/Ag interfacial modifies interfacial reflection and multilayer interference while improving interface-related charge transport, allowing the ATAA to achieve both high transmittance and low sheet resistance. In contrast, when TiN is located below Ag, the optical and electrical properties of AATA and ATATA films deteriorate markedly, and their *F_OM_* values decrease sharply to 0.36 Ω^−1^ and 0.39 Ω^−1^, respectively. Both films exhibit substantially weakened Ag (111) diffraction peaks together with pronounced reductions in carrier concentration, Hall mobility, and visible-light transmittance. Compared with AATA, ATATA shows a slight recovery in *F_OM_* after an additional TiN layer is introduced above Ag, indicating that TiN above Ag provides a compensating effect, whereas the adverse effects rising from Ag nucleation on the TiN surface still dominate. Thus, TiN regulation of the overall optoelectronic performance of the films exhibits pronounced interfacial asymmetry and stacking-sequence selectivity, with the ATAA showing the best structure–optics–electronics synergy.

## 4. Conclusions

In this study, AAA, ATAA, AATA, and ATATA multilayer films were deposited by magnetron sputtering, and the effect of the TiN interfacial layer on the microstructure and optoelectronic properties was systematically investigated. The TiN position had only a minor influence on the ZnO (002) preferred orientation and AZO grain size, but markedly affected the Ag (111) structural ordering and the overall optoelectronic performance. When TiN was located above Ag, the ATAA retained a pronounced Ag (111) diffraction feature and achieved the highest average visible-light transmittance of 85.9%, the lowest resistivity of 6.3 × 10^−5^ Ω·cm, and the highest *F_OM_* of 0.71 Ω^−1^. In contrast, when Ag grew directly on TiN, the Ag (111) diffraction peaks of AATA and ATATA films were markedly weakened, accompanied by simultaneous deterioration in optical transmittance and electrical conductivity. Compared with AATA, ATATA exhibited a slight recovery in *F_OM_* after an additional TiN layer was introduced above Ag, indicating a compensating effect that was insufficient to fully offset the adverse effect of Ag growth on TiN. Therefore, TiN at the upper and lower Ag interfaces does not play an equivalent role. The TiN-mediated regulation of AZO/Ag/AZO multilayers exhibits pronounced interfacial asymmetry and stacking-sequence selectivity. Under the present conditions, the ATAA configuration with TiN above Ag provides the best structure–optics–electronics synergy.

## Figures and Tables

**Table 1:** Deposition parameters of multilayer films.

Parameters			
Base pressure (Pa)	8.0 × 10^−4^		
Working temperature (°C)	25		
Ar flow rate (sccm)	150		
Target material	AZO	Ag	TiN
Magnetron sputtering	RF	RF	DC
Target power (W)	300	90	100
Working pressure (Pa)	1.5	1.5	0.5
Deposition time	5 min	124 s	28 s

**Table 2:** Nominal layer thickness and total nominal thicknesses of the multilayer films. The layer sequence is listed from the film surface to the glass substrate.

Film	Monolayer Thickness (nm)					Total Thickness (nm)
	AZO	TiN	Ag	TiN	AZO	
AAA	44	0	10	0	44	98
ATAA	44	2	10	0	44	100
AATA	44	0	10	2	44	100
ATATA	44	2	10	2	44	102

## Data Availability

The original contributions presented in this study are included in the article. Further inquiries can be directed to the corresponding authors.
